# Relationship between maternal obesity and prenatal, metabolic syndrome, obstetrical and perinatal complications of pregnancy in Indiana, 2008–2010

**DOI:** 10.1186/s12884-015-0696-8

**Published:** 2015-10-16

**Authors:** Shingairai A. Feresu, Yi Wang, Stephanie Dickinson

**Affiliations:** Epidemiology and Biostatistics Track, School of Health Systems and Public Health, University of Pretoria, 5-10 H.W. Snyman Building, 31 Bophelo Road, Gezina, 0031 Pretoria, South Africa; School of Health Sciences, College of Health Sciences, Walden University, 155 Fifth Ave. South, Suite 100, Minneapolis, MN 55401 USA; Department of Statistics, Indiana University, Indiana Statistical Consulting Center, 309 N Park Ave, Bloomington, IN 47405 USA

**Keywords:** Maternal prepregnancy obesity, Obesity trends, Obesity in pregnancy, Maternal complications, Medical and obstetric complications, Reproductive risk factors, Metabolic syndrorome

## Abstract

**Background:**

Obesity is a serious medical condition affecting more than 30 % of Indiana, and 25 % of Unites States pregnant women. Obesity is related to maternal complications, and significantly impacts the health of pregnant women. The objective of this study was to describe the relationship between maternal complications and pre-pregnancy maternal weight.

**Methods:**

Using logistic regression models, we analyzed 2008 to 2010 birth certificate data, for 255,773 live births abstracted from the Indiana Vital Statistics registry. We examined the risk of reproductive factors, obstetrical complications and perinatal (intrapartum) complications for underweight, healthy weight, overweight and obese women for this population.

**Results:**

Women who received prenatal care were more likely to be obese [adjusted odds ratio (AOR) = 1.82 (1.56–2.13)]. While women with parity of zero (0) were less likely to be obese [AOR = 0.89, 95 % CI (0.86–0.91)]. Women giving birth to twins [AOR = 1.25, 95 % CI (1.17– 1.33)], women delivering by Caesarian section [AOR = 2.31, 95 % CI ( 2.26–2.37)], and women who previously had a Caesarian section [AOR = 1.95, 95 % CI (1.88–2.02)] were more likely to be obese. There was evidence of metabolic like complication in this population, due to obesity. Obesity was significantly associated with obstetrical conditions of the metabolic syndrome, including pre-pregnancy diabetes, gestational diabetes, pre-pregnancy hypertension, pregnancy-induced hypertension and eclampsia [AOR = 5.12, 95 % CI (4.47–5.85); AOR = 3.87, 95 % CI (3.68–4.08); AOR = 7.66, 95 % CI (6.77–8.65); AOR = 3.23, 95 % CI (3.07–3.39); and AOR = 1.77, 95 % CI (1.31–2.40), respectively. Maternal obesity modestly increased the risk of induction, epidural, post-delivery bleeding, and prolonged labor [AOR = 1.26, 95 % CI (1.23–1.29); AOR = 1.15, 95 % CI (1.13–1.18); AOR = 1.20, 95 % CI (1.12–1.28); and AOR = 1.44, 95 % CI (1.30–1.61)], respectively. Obese women were less likely to have blood transfusions [AOR = .74, 95 % CI (0.58–96)], vaginal tears [AOR = 0.51, 95 % CI (0.44–0.59)], or infections [AOR = 86, 95 % CI (0.80–0.93)].

**Conclusions:**

Our results suggest that maternal obesity in Indiana, like other populations in the USA, is associated with high risks of maternal complications for pregnant women. Pre-pregnancy obesity prevention efforts should focus on targeting children, adolescent and young women, if the goal to reduce the risk of maternal complications related to obesity, is to be reached.

## Background

Obesity and overweight, defined as a body mass index (BMI) ≥30 kg/m^2^ and BMI ≥ 25 kg/m^2^, respectively [1–4], continue to affect the health of pregnant women globally, but particularly for developed nations [5]. BMI is measured as body weight (kg) divided by height (meters) squared [1–4]. The consequences of obesity have been costly [6, 7], and challenging for health care, as more and more people are impacted by the complications related to obesity [1, 4, 7], and particularly for obstetric care [8–14]. The prevalence of obesity has risen, particularly in developed countries due to sedentary lifestyles [5, 15, 16]. The number of women, especially pregnant women, who are overweight or obese, has become increasingly common [1, 4, 5, 9, 12, 17]. The prevalence of obesity among pregnant women in the United States (US) was reported as 29.1 % for 2010 [1], and we report a prevalence of 23 % for Indiana pregnant women for 2010 in our previous study (in review).

Obesity was recognized as a risk factor in pregnancy more than 50 years ago [5, 9], and as rates have increased, it has continued to complicate obstetric and maternity care [4, 8–14, 18, 19]. Obesity has been associated with greater risk of infertility, maternal morbidity, and complications of labor and delivery [4, 8–14, 18, 19]. In early pregnancy there is an increased risk of spontaneous abortion and congenital anomalies [12, 20]. In later gestation, gestational hypertensive disorders (preeclampsia, eclampsia) and diabetes are clinically recognized, which present metabolic like complication of pregnancy in obese women [5, 9–14, 18, 20–23]. At birth, macrosomia has been postulated to result in maternal complications leading to induction of labor, cesarean section, and complications of anesthesia [4, 5, 13, 14, 18, 21]. At postpartum, obesity is also associated with increased risk of deep venous thrombophlebitis, postpartum hemorrhage, and infectious morbidity [5, 9–14, 18, 20–23]. For Indiana, obesity is costly, and medical costs are projected to increase to $7 billion by 2018 [24]. While obesity in pregnancy has been heavily studied in the last decade on a national level in the USA, with the latest results reported in 2012 [17], state specific publications have been scanty. There is limited data which examines the relationship between maternal complications of pregnancy and maternal pre-pregnancy weight for Indiana. Such data on the state level is important for policy and intervention strategies. Given this situation, our objective is to describe the relationship between maternal obesity and maternal complications of pregnancy for women delivering a liveborn infant in Indiana from 2008 to 2010.

## Methods

We analyzed birth certificate data for live births from the Indiana State Department of Health (ISDH) abstracted from the Vital Statistics registry on all births occurring in Indiana from 2008 through 2010, with complete data on body mass index (BMI) of the mother (*N* = 255,773). The BMI was calculated from self-reported pre-pregnancy weight and height of the pregnant women in this dataset. Socio-demographic data for mothers delivering a liveborn infant are described in our previous paper (in review), along with average BMI, and rates of overweight and obesity for each demographic group. In this current paper we selected variables for reproductive factors, obstetrical complications, and maternal perinatal complications. The average BMI was calculated for women in each level of the reproductive factors, obstetrical and perinatal complications, and with the frequency and percentage of women in each weight group: underweight (BMI < 18.5), normal weight (BMI 18.5 to 24.9), overweight (BMI 25 to 29.9), and obese (BMI 30+).

Reproductive factors included prenatal care (yes/no), parity (0, 1, 2, >2), gestation type (singleton, twins, triplets, more than 3), delivery type (vaginal, caesarian, vacuum, forceps), previous caesarian section delivery (yes/no), and previous preterm birth (yes/no). The percent of women in each BMI group (underweight, healthy weight, overweight, and obese) were calculated for each reproductive factor, and odds ratios were calculated comparing overweight, or obese, to healthy weight for each reproductive factor. Binary logistic regression models were performed with BMI group as the outcome variable (overweight vs healthy and obese vs healthy) and each reproductive factor as the predictor variable. Adjusted odds ratios were calculated by controlling for mother’s age, race, ethnicity, marital status, maternal education, paternal education, and smoking.

Obstetrical complications analyzed included prepregnancy diabetes, gestational diabetes prepregnancy hypertension, gestational hypertension, eclampsia, and placenta abruption. Maternal perinatal complications analyzed were induction, blood transfusion, epidural, post-delivery bleeding, prolonged labor, 3^rd^ to 4^th^ degree vaginal tear, chorioamnionitis, infection, and whether or not steroids were used. The percent of women having each complication were calculated for each weight group. Odds Ratios were calculated with logistic regression models to estimate the odds of having each individual obstetrical or perinatal maternal complication, with predictor variables for underweight, overweight and obese women using healthy weight women as the reference group. Adjusted odds ratios were also calculated, controlling for mother’s age, race, ethnicity, marital status, maternal education, paternal education, and smoking. Analyses were performed using SAS/STAT software, version 9.3 of the SAS System for Windows.

Permission to use the data was obtained from Indiana State Department of Health, Public Health Protection and Laboratory Services Commission, Epidemiology Resource Center, and was provided without personal identifying information under strict conditions. The study was approved by the Indiana University Institutional Review Board (IRB) IRB STUDY NUMBER: 1104005136.

## Results

### Reproductive factors of pregnancy

Table 1 presents the distribution of mothers’ BMI for each reproductive factor. Prenatal care was very high, such that 98.9 % of women giving birth received prenatal care. About 40 % of the women delivered their first child, and just over 3 % delivered multiple births (twins, triplets, or more). About 66 % of the births were delivered vaginally, while 30 % were delivered by Caesarian section. Less than 12 % of the women had previously delivered by Caesarian section.

**Table 1:** Distribution of weight by reproductive factors of pregnancy for women (*n* = 255,773) delivering in Indiana 2008 through 2010

			Underweight	Healthy weight	Overweight	Obese
	Total	BMI	Below 18.5	18.5 to 24.9	25 to 29.9	30 and above
	*n* (%)	M (SD)	*n* (%)	*n* (%)	*n* (%)	*n* (%)
Prenatal care^a^						
Yes	2,4936,4 (98.9)	26.5 (6.7)	6558 (2.6)	103,903 (41.7)	81,921 (32.9)	56,982 (22.9)
No	2807 (1.1)	25.5 (6.4)	141 (5.0)	1269 (45.2)	900 (32.1)	497 (17.7)
Parity^b^						
0	101,170 (39.6)	25.9 (6.5)	3154 (3.1)	46,621 (46.1)	31,255 (30.9)	20,140 (19.9)
1	79,228 (31.0)	26.7 (6.8)	1988 (2.5)	32,088 (40.5)	26,475 (33.4)	18,677 (23.6)
2	43,800 ( 17.1)	27.0 (6.8)	989 (2.3)	16,779 (38.3)	15,080 (34.4)	10,952 (25.0)
>2	31,404 (12.3)	27.4 (6.9)	690 (2.2)	11,168 (35.6)	11,086 (35.3)	8460 (26.9)
Gestation type^c^						
Singleton	2,473,84 (96.7)	265 (6.7)	6658 (2.7)	103,542 (41.9)	81,093 (32.8)	56,091 (22.7)
Twins	7927 (3.1)	273 (7.1)	167 (2.1)	2984 (37.6)	2707 (34.2)	2069 (26.1)
Triplets	392 (0.2)	271 (7.0)	3 (0.8)	165 (42.1)	134 (34.2)	90 (23.0)
More than 3	67 (0.0)	21.4 (5.9)	33 (49.3)	17 (25.4)	8 (11.9)	9 (13.4)
Delivery type^d^						
Vaginal	1,671,00 (65.8)	25.8 (6.2)	4,937 (3.0)	75,762 (45.3)	54,710 (32.7)	31,691 (19.0)
Caesarian	76,57 (30.2)	28.3 (7.5)	1443 (1.9)	25,103 (32.8)	25,563 (33.4)	24,548 (32.0)
Vacuum	8774 (3.5)	25.1 (6.0)	327 (3.7)	4365 (49.8)	2697 (30.7)	1385 (15.8)
Forceps	1519 (0.6)	25.4 (6.2)	54 (3.6)	742 (48.9)	471 (31.0)	252 (16.6)
Previous Caesarian section delivery^e^						
Yes	29,812 (11.7)	28.6 (7.5)	487 (1.6)	9216 (30.9)	10264 (34.4)	9845 (33.0)
No	2,259,21 (88.3)	26.2 (6.5)	6341 (2.8)	97,491 (43.2)	73,677 (32.6)	48,412 (21.4)

^a^3,602 observations had missing information on prenatal care

^b^171 observations had missing information on parity

^c^3 observations had missing information on gestation type

^d^1,723 observations had missing information on delivery type

^e^40 observations had missing information on previous Caesarian section

The crude and adjusted odds ratios for reproductive factors by pre-pregnancy BMI of the mother are presented in Table 2. Women who received prenatal care were more likely to be overweight [adjusted odds ratio (AOR) = 1.28 95 % CI (1.14–1.45) or obese AOR = 1.82 (1.56–2.13)]. Women with no previous births were less likely to be overweight or obese [AOR = 0.89, 95 % CI (0.87–0.91), AOR = 0.89, 95 % CI (0.86–0.91), for overweight and obese, respectively]. Also, women who delivered by instrumentation (vacuum, or forceps) were less likely to be overweight or obese. In crude analysis, women who were overweight or obese were more likely to have parity of 2, but not significantly after adjusting for other demographics. Overweight and obesity were more common among women delivering twins [AOR = 1.11, 95 % (1.05–1.18) for overweight, AOR = 1.25, 95 % (1.17–1.33) for obesity], women delivering by Caesarian section [AOR = 1.40, 95 % CI (1.37–1.43) for overweight, and AOR 2.31, 95 % CI (2.26–2.37) for obesity], and women who previously delivered by Caesarian section [AOR = 1.38, 95 % CI (1.33–1.42); for overweight, and AOR = 1.95, 95 % CI (1.88–2.02) for obesity].

**Table 2:** Crude and adjusted^a^ odds ratios for overweight and obesity, compared to healthy weight, by reproductive factors of pregnancy for women delivering in Indiana 2008 through 2010 (*n* = 255,773)

	Overweight crude	Overweight adjusted^a^	Obesity crude	Obesity adjusted^a^
	Odds ratio (95 % CI)^b^	Odds ratio (95 % CI)	Odds ratio (95 % CI)	Odds ratio (95 % CI)
Prenatal care				
Yes	1.11 (1.02–1.20)	1.28 (1.14–1.45)	1.41 (1.27–1.56)	1.82 (1.56–2.13)
No	Reference	Reference	Reference	Reference
Parity				
0	0.81 (0.80– 0.83)	0.89 (0.87– 0.91)	0.74 (0.72–0.76)	0.89 (0.86–0.91)
1	Reference	Reference	Reference	Reference
2	1.09 (1.06–1.12)	1.01 (0.98–1.04)	1.12 (1.09–1.16)	1.00 (0.97–1.04)
>2	1.20 (1.17–1.24)	1.05 (1.02–1.09)	1.30 (1.26–1.35)	1.02 (0.98-1.06)
Gestation type^c^				
Singleton	Reference	Reference	Reference	Reference
Twins	1.16 (1.10–1.22)	1.11 (1.05–1.18)	1.28 (1.21–1.35)	1.25 (1.17–1.33)
Triplets	1.04 (0.82–1.30)	1.10 (0.87–1.39)	1.01 (0.78–1.30)	1.15 (0.87–1.52)
More than 3	0.60 (0.26–1.39)	0.79 (0.34–1.84)	0.98 (0.44–2.19)	1.06 (0.38–2.96)
Delivery type				
Vaginal	Reference	Reference	Reference	Reference
Caesarian	1.41 (1.38–1.44)	1.40 (1.37–1.43)	2.34 (2.29–2.39)	2.31 (2.26–2.37)
Vacuum	0.86 (0.81–0.90)	0.90 (0.85–0.95)	0.76 (0.71–0.81)	0.81 (0.76–0.87)
Forceps	0.88 (0.78–0.99)	0.92 (0.81–1.05)	0.81 (0.70–0.94)	0.87 (0.74–1.02)
Previous Caesarian section delivery				
Yes	1.47 (1.43–1.52)	1.38 (1.33–1.42)	2.15 (2.09–2.22)	1.95 (1.88–2.02)
No	Reference	Reference	Reference	Reference

^a^adjusted for age, race, ethnicity, marital status, maternal education, paternal education, smoking,

^b^CI = confidence intervals

^c^refers to current pregnancy

### Obstetrical complications of pregnancy

Overall, the percentage of women with maternal obstetrical complications in Indiana was very small: prepregnancy diabetes (0.8 %), gestational diabetes (4.9 %) prepregnancy hypertension (1.1 %), gestational hypertension (5.3 %), eclampsia (0.1 %), or abruptio placenta (0.5 %) (Table 3). The mean BMI for women with obstetrical complications ranged from 26.1 for women with abruption placenta, to 34.1 for women with prepregnancy hypertension. For overweight women, the highest percentages were for gestational hypertension (5.4 %) and for gestational diabetes (4.9 %). The percentages were higher for obese women for gestational hypertension and diabetes (9.3 % and 9.5 %, respectively). The percentage of obstetrical complications were small for underweight and healthy weight women, with the highest percentage of only 2.3 % for gestational hypertension and 2.1 % for gestational diabetes for underweight women, and 3.2 % and 2.5 % for gestational hypertension and diabetes, respectively, for healthy weight women.

**Table 3:** Distribution of Weight by Obstetrical Complications of Pregnancy for Women Delivering in Indiana 2008 through 2010 (*n* = 255,773)

				Underweight	Healthy weight	Overweight	Obese
	Total	BMI		Below 18.5	18.5 to 24.9	25 to 29.9	30 and above
	*n* %	M (SD)	Median	*n* (%)	*n* (%)	*n* (%)	*n* (%)
Prepregnancy diabetes^a^							
Yes	2036 (0.8)	32.8 (8.9)	31.5	20 (0.3)	327 (0.3)	559 (0.7)	1130 (1.9)
No	253,697 (99.2)	26.5 (6.7)	25.2	6808 (99.7)	1,063,80 (99.7)	83,382 (99.3)	57,127 (98.1)
Gestational diabetes^b^							
Yes	12,488 (4.9)	30.5 (7.9)	29.4	141 (2.1)	2685 (2.5)	4124 (4.9)	5538 (9.5)
No	243,245 (95.1)	26.3 (6.6)	25.2	6687 (97.9)	1,040,22 (97.5)	79,817 (95.1)	52,719 (90.5)
Prepregnancy hypertension^c^							
Yes	2892 (1.1)	34.1 (9.1)	33.6	22 (0.3)	385 (0.4)	708 (0.8)	1777 (3.0)
No	252,841 (98.9)	26.4 (6.6)	25.2	6806 (99.7)	106,322 (99.6)	83,233 (99.2)	56,480 (97.0)
Gestational hypertension^d^							
Yes	13,474 (5.3)	29.9 (7.9)	27.3	157 (2.3)	3372 (3.2)	4511 (5.4)	5434 (9.3)
No	242,259 (94.7)	26.3 (6.6)	25.2	6671 (97.7)	1,033,35 (96.8)	79,430 (94.6)	52,823 (90.7)
Eclampsia^e^							
Yes	350 (0.1)	28.1 (6.7)	27.3	6 (0.1)	99 (0.1)	137 (0.2)	108 (0.2)
No	255,246 (99.9)	26.5 (6.7)	25.2	6817 (99.9)	1,065,50 (99.9)	83,762 (99.8)	58,117 (99.8)
Abruptio placenta^f^							
Yes	1274 (0.5)	26.1 (6.7)	25.2	52 (0.8)	555 (0.5)	388 (0.5)	279 (0.5)
No	252,210 (99.5)	26.5 (6.7)	25.2	6693 (99.2)	1,051,57 (99.5)	82,881 (99.5)	57,479 (99.5)

^a^40 observations had missing information on prepregnancy diabetes

^b^40 observations had missing information on gestational diabetes

^c^40 observations had missing information on prepregnancy hypertension

^d^40 observations had missing information on gestational hypertension (pre-eclampsia)

^e^177 observations had missing information on eclampsia

^f^2,289 observations had missing information on abruptio placenta

Women who were overweight or obese were more likely to have prepregnancy diabetes, gestational diabetes, prepregnancy hypertension, gestational hypertension, and eclampsia in crude and adjusted analysis (Table 4). The strongest risks were for prepregnancy hypertension [AOR = 7.66, 95 % CI (6.77–8.65) and for prepregnancy diabetes, AOR = 5.12, 95 % CI (4.47–5.85)], respectively, in obese women. Underweight women were more likely to have abruptio placenta [AOR = 1.40, 95 % CI (1.01–1.94)], but less likely to have gestational hypertension [AOR = 0.75, 95 % CI (0.63–0.90)], and also gestational diabetes, only in crude analysis.

**Table 4:** Crude and adjusted^a^ odds ratios of obstetrical complications for underweight, overweight, and obese women compared to normal weight, for women delivering in Indiana 2008 through 2010 (*n* = 255,773)

	Under weight		Overweight		Obesity	
	Crude OR^c^(95 % CI^d^)	Adjusted^a^ OR (95 % CI)	Crude OR (95 % CI)	Adjusted^a^ OR (95 % CI)	Crude OR (95 % CI)	Adjusted^a^ OR (95 % CI)
Prepregnancy diabetes	0.96 (0.61–1.50)	1.02 (0.62–1.66)	2.18 (1.90–2.50)	1.84 (1.59–2.14)	6.44 (5.69–7.28)	5.12 (4.47–5.85)
Gestational diabetes	0.82 (0.69–0.97)	0.90 (0.75–1.09)	2.00 (1.91–2.10)	1.90 (1.80–2.00)	4.07 (3.88–4.27)	3.87 (3.68–4.08)
Prepregnancy Hypertension	0.89 (0.58–1.37)	1.02 (0.62–1.66)	2.35 (2.07–2.66)	2.27 (1.99–2.60)	8.69 (7.78–9.71)	7.66 (6.77–8.65)
Gestational Hypertension^b^	0.72 (0.61–0.85)	0.75 (0.63–0.90)	1.74 (1.66–1.82)	1.80 (1.71–1.89)	3.15 (3.02–3.30)	3.23 (3.07–3.39)
Eclampsia	0.95 (0.42–2.16)	0.74 (0.27–2.02)	1.76 (1.36–2.28)	1.54 (1.16–2.05)	2.00 (1.52–2.63)	1.77 (1.31–2.40)
Abruptio placenta	1.47 (1.11–1.96)	1.40 (1.01–1.94)	0.89 (0.78–1.01)	0.89 (0.76–1.03)	0.92 (0.80–1.06)	0.98 (0.83–1.15)

^a^Reference group = normal weight (BMI 18.5 to 24.9), adjusted for age, race, ethnicity, marital status, maternal education, paternal education, smoking

^b^Pre-eclampsia

^c^OR = odds ratio

^d^CI = confidence intervals

### Perinatal (intrapartum) complications

The overall percentages for maternal perinatal complications were highest for epidural (67.8 %), and induction (31.4 %), and negligible with percentages below 1 % for blood transfusion, chorioamnionitis, and vaginal tears (Table 5). The mean BMI for all the perinatal complications were between 24.5 and 27.4. The distributions for perinatal complications were similar for overweight and obese women. In crude and adjusted analysis, overweight and obese women were more likely to have induction, epidural, post-delivery bleeding and prolonged labor (Table 6). Overweight and obese women were less likely to have vaginal tears and infections, in both crude and adjusted analysis. Overweight women were less likely to be on steroids, while obese women were less likely to have chorioamnionitis and more likely to be on steroids in crude analysis only. Underweight women were less likely to have induction, but were more likely to have blood transfusion, steroids, and infections (in crude analysis only).

**Table 5:** Distribution of overweight and obesity by maternal perinatal complications for women (*n* = 255,773) delivering in Indiana 2008 through 2010

				Underweight	Healthy weight	Overweight	Obese
	Total	BMI		Below 18.5	18.5 to 24.9	25 to 29.9	30 and above
	*n* (%)	M (SD)	Median	*n* (%)	*n* (%)	*n* (%)	*n* (%)
Induction^a^							
Yes	80,221 (31.4)	26.9 (6.8)	25.2	1890 (27.7)	31,670 (29.7)	26,909 (32.1)	19,752 (33.9)
No	1,755,17 (68.6)	26.4 (6.7)	25.2	4938 (72.3)	75,039 (70.3)	57,033 (67.9)	38,507 (66.1)
Blood transfusion^b^							
Yes	553 (0.2)	25.7 (6.4)	25.2	28 (0.4)	247 (0.2)	170 (0.2)	108 (0.2)
No	2,551,80 (99.8)	26.5 (6.7)	25.2	6800 (99.6)	106,461 (99.8)	83,770 (99.8)	58,149 (99.8)
Epidural^c^							
Yes	1,733,22 (67.8)	26.5 (6.7)	25.2	4549 (66.62)	71,898 (67.4)	57,189 (68.1)	39,686 (68.1)
No	82,416 (32.2)	26.5 (6.7)	25.2	2279 (33.38)	34,811 (32.6)	26,753 (31.9)	18,573 (31.9)
Post-delivery bleeding^d^							
Yes	8505 (3.3)	27.0 (6.9)	25.2	204 (3.0)	3332 (3.1)	2837 (3.4)	2132 (3.7)
No	247,233 (96.7)	26.5 (6.7)	25.2	6624 (97.0)	1,033,77 (96.9)	81,105 (96.6)	56,127 (96.3)
Prolonged labor^e^							
Yes	2707 (1.1)	27.4 (7.4)	25.2	63 (0.9)	1033 (1.0)	879 (1.0)	732 (1.3)
No	2,530,59 (98.9)	26.5 (6.7)	25.2	6798 (99.1)	1,056,75 (99.0)	83,060 (99.0)	57,526 (98.7)
3^rd^ to 4^th^ degree vaginal tear^f^							
Yes	2076 (0.8)	24.5 (5.3)	23.1	61 (0.9)	1115 (1.0)	640 (0.8)	260 (0.4)
No	2,536,57 (99.2)	26.5 (6.7)	25.2	6767 (99.1)	1,055,93 (99.0)	83,300 (99.2)	57,997 (99.6)
Chorioamnionitis^g^							
Yes	1784 (0.7)	26.1 (6.2)	25.2	40 (0.6)	778 (0.7)	601 (0.7)	365 (0.6)
No	2,539,54 (99.3)	26.5 (6.7)	25.2	6788 (99.4)	1,059,31 (99.3)	83,341 (99.3)	57,894 (99.4)
Infection^h^							
Yes	8789 (3.4)	26.1 (6.7)	25.2	337 (4.9)	3827 (3.6)	2751 (3.3)	1874 (3.2)
No	2,469,84 (96.6)	26.5 (6.7)	25.2	6524 (95.1)	1,028,83 (96.4)	81,191 (96.7)	56,386 (96.8)
Steroids^i^							
Yes	4430 (1.7)	26.8 (7.4)	25.2	155 (2.3)	1835 (1.7)	1306 (1.6)	1134 (1.9)
No	251,308 (98.3)	26.5 (6.7)	25.2	6673 (97.7)	1,048,74 (98.3)	82,636 (98.4)	57,125 (98.1)

^a^35 observations had missing information on Induction

^b^40 observations had missing information on blood transfusion

^c^35 observations had missing information on epidural

^d^35 observations had missing information on post delivery bleeding

^e^7 observations had missing information on prolonged labor

^f^40 observations had missing information on 3rd and 4th degree vaginal tear

^g^35 observations had missing information on chorioamnionitis

^h^No observations had missing information on infection

^i^35 observations had missing information on steroids

**Table 6:** Crude and adjusted^a^ odds ratios for overweight and obesity by maternal perinatal complications for women (n = 255,773) delivering in Indiana 2008 through 2010

	Under weight		Overweight		Obesity	
	Crude OR^b^(95 % CI^c^)	Adjusted^a^ OR (95 % CI)	Crude OR (95 % CI)	Adjusted^a^ OR (95 % CI)	Crude OR^b^(95 % CI^†^)	Adjusted^a^ OR (95 % CI)
Induction	0.91 (0.86–0.96)	0.88 (0.82–0.93)	1.12 (1.10–1.14)	1.17 (1.15–1.20)	1.22 (1.19–1.24)	1.26 (1.23–1.29)
Blood transfusion	1.78 (1.20–2.63)	1.97 (1.29–2.99)	0.88 (0.72–1.06)	0.87 (0.70–1.08)	0.80 (0.64–1.00)	0.74 (0.58–0.96)
Epidural	0.97 (0.92–1.02)	0.97 (0.92–1.03)	1.04 (1.02–1.06)	1.11 (1.09–1.14)	1.04 (1.01–1.06)	1.15 (1.13–1.18)
Post–delivery bleeding	0.96 (0.83–1.10)	0.92 (0.97–1.16)	1.09 (1.03–1.14)	1.07 (1.01–1.14)	1.18 (1.12–1.25)	1.20 (1.12–1.28)
Prolonged labor	0.95 (0.73–1.22)	0.94 (0.71–1.25)	1.08 (0.99–1.19)	1.14 (1.03–1.26)	1.30 (1.18–1.43)	1.44 (1.30–1.61)
3^rd^ to 4^th^ vaginal tear	0.85 (0.66–1.11)	0.95 (0.71–1.26)	0.73 (0.66–0.80)	0.82 (0.73–0.90)	0.43 (0.37–0.49)	0.51 (0.44–0.59)
Chorioamnionitis	0.80 (0.58–1.10)	0.87 (0.61–1.24)	0.98 (0.88–1.09)	1.03 (0.91–1.16)	0.86 (0.76–0.97)	0.94 (0.82–1.09)
Infection	1.39 (1.24–1.56)	1.04 (0.89–1.21)	0.91 (0.87– 0.96)	0.92 (0.86–0.98)	0.89 (0.85–0.95)	0.86 (0.80–0.93)
Steroids	1.33 (1.13–1.57)	1.35 (1.11–1.63)	0.90 (0.84–0.97)	0.85 (0.78–0.92)	1.14 (1.05–1.22)	1.07 (0.98–1.16)

^a^Reference group = normal weight (BMI 18.5 to 24.9), adjusted for age, race, ethnicity, marital status, maternal education, paternal education, smoking

^b^OR = odds ratio

^c^CI = confidence intervals

## Discussion

In this study we describe the relationship between BMI groups (underweight, overweight, and obesity) compared to healthy weight women for each reproductive factor, and maternal obstetrical and perinatal complication of pregnancy for women delivering in Indiana from 2008 to 2010. Our findings support the role of obesity with regards to increasing the metabolic-like complications in particular.

### Reproductive factors of pregnancy

Overweight and obese women were more likely to attend prenatal care. Some recent studies found no difference between the use of prenatal care for obese versus no-obese women [25], and also that obesity was not an independent barrier to receiving early and adequate prenatal care [26]. Recommendations for care of overweight and obese women are focused on the use of prenatal care as a way to intervene, and reduce excessive weight gain during pregnancy for pregnant women [8, 19, 27].

Overweight and obese women were less likely to be parity of 0, but were more likely to be of parity 2 and more in our study. The association between high parity and maternal obesity is plausible as was reported by other studies in many different countries [20, 28–38]. Multiparous women may have gained weight as a result of their increased food intake, reduced physical activity, or both, or it may have been due to the effect of possible persistent changes in endocrine functions following repeated pregnancies [38]. Studies report the relationship between metabolic syndrome (of which maternal obesity is a part) and high parity and gravida [20–22, 28]. The relationship between parity and prepregnancy obesity, particularly with regards to waist circumference and BMI, may be dependent on race [34, 36, 37]. In our study, the percentage of women with high parity (≥2) was 28.66 % for white women, 35.64 % for Black, and 39.19 % for Hispanic compared to non-Hispanic women. This relationship between parity and prepregnancy obesity has been described by Kim et. al 2007 as related to country level of development [34]. Mishra 2007, in her commentary, describes this relationship as part of life course experience [35].

Our results that overweight and obese women were more likely to deliver twins, is in agreement with other studies [39, 40].

### Obstetrical complications of pregnancy

As expected, overweight and obese women were more likely to have maternal metabolic syndrome-like obstetrical complications, as described by other literature [28, 41, 42]. These complications, of which obesity is a part, include pre-pregnancy diabetes, gestational diabetes, pre-pregnancy hypertension, gestational hypertension, and eclampsia, particularly in late pregnancy. Metabolic syndrome and metabolic risk factors were defined according to the standard criteria of the National Cholesterol Education Program’s Adults Treatment Panel III (NCEP-ATP III) [43]. “Three or more of the following components constituted metabolic syndrome: a) abdominal obesity, as measured by a waist circumference of ≥ 88 cm for women; b) high fasting blood glucose (≥110 mg/dL or ≥6.1 mmol/L) or patients diagnosed with diabetes; c) high triglycerides (≥150 mg/dL or ≥1.7 mmol/L); d) low HDL cholesterol (<50 mg/dL or <1.29 mmol/L); e) and high blood pressure (≥130/≥85 mmHg)” [28, 43].

The relationship between obesity and prepregnancy diabetes as confirmed by our study, is also part of metabolic syndrome, or insulin resistance, common to people with central obesity, and has been described by other studies [4, 5, 9, 12, 13, 20–23, 31]. In some instances, prepregnancy diabetes may be undiagnosed [12]. Women with prepregnancy diabetes usually enter pregnancy with obesity and Type II diabetes [12, 21–23, 43]. Our findings that overweight and obese women were more likely to have gestational diabetes, is also in agreement with other studies [4, 5, 9–14, 19–23]. Gestational diabetes may follow insulin resistance, a manifestation of obesity [12, 43]. On the other hand, obesity could trigger insulin resistance in some cases via increased C-reactive protein (CRP) levels in late pregnancy, when it may relate to pregravid BMI and not to gestational diabetes mellitus [42, 43].

We found that overweight and obese women were more likely to have prepregnancy hypertension, as reported by other studies [4, 9, 10, 20–22, 30, 44]. This is in part due to the metabolic syndrome. Overweight and obese Indiana pregnant women were more likely to have gestational hypertension or pregnancy-induced hypertension, or preeclampsia in agreement with other studies [4, 9, 14, 18, 20–23, 44–49], and eclampsia as documented by other studies [9, 14, 20–23]. These findings are plausible as this is another demonstration of the metabolic syndrome which is due, in part to obesity. In our study though, about 0.58 %.of women had both prepregnancy diabetes and prepregnancy hypertension. Only 0.08 % of women in our study had both gestational diabetes and gestational hypertension, show-casing the independent effect of each disease that makes up this syndrome in pregnant women, who are obese. It is possible that this is an underestimation of the overlapping representation of obesity, diabetes and hypertension. The likelihood that only the primary diagnosis was considered in the data, is plausible. Conversely, underweight women were less likely to have gestational hypertension and gestational diabetes, however, the later was observed only in crude analysis.

### Perinatal (intrapartum) complications

Obese and overweight women were more likely to deliver by Caesarian section, or to have a history of Caesarian section. Delivery by Caesarian section for overweight and obese women is consistent with other studies [4, 9–11, 14, 18, 22, 30, 50–57]. Some studies attribute this relationship to macrosomic necessitating a Caesarian section [18, 22, 51, 52], while other report independent relationship between maternal obesity and Caesarian section [4, 14, 50, 52, 55–57]. Studies have also documented that obesity necessitates an emergency Caesarian section [10, 18, 50]. Chu 2007 et al., in their review explain that the biological pathway through which obesity affects the labour process is not well understood [52]. They also argue that “some studies have suggested that obesity increases maternal pelvic soft tissue which narrows the diameters of the birth canal and increases the risk of Caesarian section delivery, which is associated with dystocia, a macrosomic infant, or cephalopelvic disproportion [52], while others have suggested that the increased risk of Caesarean deliveries could be related to differences in labour progression among obese women or their response to oxytocin administration” [52].

Overweight and obese women were less likely to deliver by instrumentation (vacuum and forceps), as was reported by other studies [10]. Some studies found that Class III obesity was associated with operative vaginal delivery [18, 20, 22]. Our study found that overweight and obese women were more likely to have induction, a finding reported by other studies [4, 9, 10, 14, 20–22, 30, 58]. In some studies, induction in obese women was minimal, and did not affect management [58]. In contrast, underweight women were less likely to have induction, confirming the role obesity plays during the intrapartum period. Overweight and obese were more likely to have epidural, probably due to prolonged labor. This is not surprising, as epidural is a recommended treatment protocol for obese women [8, 19, 27].

Also, overweight and obese women were more likely to have post-delivery bleeding and prolonged bleeding, consistent with other studies [4, 9, 11, 22, 30], but contradicts other studies [10]. Some studies [11, 59], also suggest that high pre-pregnancy BMI substantially increased the risk of postpartum anemia, probably as a consequence in part of post-delivery bleeding and prolonged bleeding.

Studies have reported that overweight and obese women were less likely to have 3^rd^ to 4^th^ degree tear [10]. Interestingly, overweight and obese women, in our study were less likely to have an infection and chorionamnionitis in particular, unlike other studies, where infection plays a significant role [20–22]. Also overweight women were less likely to use steroids, while obese women were more likely to use steroids. The role of infection for obese pregnant women needs further exploration.

### Strengths and limitations

A few limitations warrant mention. We could only analyze data which the state made available to us, which they felt would not violate protection of personal information for the women delivering in Indiana. In assessing whether there was an overlap between obesity, diabetes and hypertension, which acts in concert for the metabolic syndrome, the percentage overlap was minimal, possibly because only the primary diagnosis may have been entered for the dataset, thus underestimating this phenomenon. Using self-reported data could introduce misclassification for obesity. The validity of using self-reported weight being concern in obesity studies, particularly with underreporting of prepregnancy weight has been reported by other studies [60–62]. These studies report that there is an underreporting of obesity in self-reported weight and height of between 3–5 % [60–62], which would result in an underestimate of our estimates. However a more recent study [63], argues that misclassification from self-reported weight and height has a minimal and limited impact on reliability and validity for population-based surveillance and research purposes.

Nonetheless, our study is the first to describe the relationship between maternal complications of pregnancy and prepregnancy obesity for Indiana. It is important that prepregnancy obesity is well understood, screening undertaken, and better management [8, 12], as children born to overweight and obese women, compared to those of normal weight women, have greater risk for becoming obese themselves and suffering comorbid metabolic health problems [42].

## Conclusion

Our study confirms that metabolic complications, as well as other prenatal, perinatal and obstetrical complications are associated with pre-pregnancy obesity. While several studies have described these associations, in different populations, no such studies exist for the state of Indiana. A more targeted approach at state level will help with targeted interventions. Thus, such findings add to the existing body of literature, and are valuable to states like Indiana.

Our results suggest that maternal obesity in Indiana, like in other populations in the USA, is associated with high risks of maternal complications for pregnant women. Pre-pregnancy obesity prevention efforts should focus on targeting children, adolescent and young women, if the goal to reduce the risk of maternal complications related obesity, is to be reached.
